# Identification of Novel Single Nucleotide Polymorphisms Associated with Acute Respiratory Distress Syndrome by Exome-Seq

**DOI:** 10.1371/journal.pone.0111953

**Published:** 2014-11-05

**Authors:** Katherine Shortt, Suman Chaudhary, Dmitry Grigoryev, Daniel P. Heruth, Lakshmi Venkitachalam, Li Q. Zhang, Shui Q. Ye

**Affiliations:** 1 Department of Pediatrics, Division of Experimental and Translational Genetics, Children's Mercy Hospital, University of Missouri - Kansas City School of Medicine, Kansas City, Missouri, United States of America; 2 Department of Biomedical and Health Informatics, University of Missouri - Kansas City School of Medicine, Kansas City, Missouri, United States of America; University of Illinois College of Medicine, United States of America

## Abstract

Acute respiratory distress syndrome (ARDS) is a lung condition characterized by impaired gas exchange with systemic release of inflammatory mediators, causing pulmonary inflammation, vascular leak and hypoxemia. Existing biomarkers have limited effectiveness as diagnostic and therapeutic targets. To identify disease-associating variants in ARDS patients, whole-exome sequencing was performed on 96 ARDS patients, detecting 1,382,399 SNPs. By comparing these exome data to those of the 1000 Genomes Project, we identified a number of single nucleotide polymorphisms (SNP) which are potentially associated with ARDS. 50,190SNPs were found in all case subgroups and controls, of which89 SNPs were associated with susceptibility. We validated three SNPs (rs78142040, rs9605146 and rs3848719) in additional ARDS patients to substantiate their associations with susceptibility, severity and outcome of ARDS. rs78142040 (C>T) occurs within a histone mark (intron 6) of the Arylsulfatase D gene. rs9605146 (G>A) causes a deleterious coding change (proline to leucine) in the XK, Kell blood group complex subunit-related family, member 3 gene. rs3848719 (G>A) is a synonymous SNP in the Zinc-Finger/Leucine-Zipper Co-Transducer NIF1 gene. rs78142040, rs9605146, and rs3848719 are associated significantly with susceptibility to ARDS. rs3848719 is associated with APACHE II score quartile. rs78142040 is associated with 60-day mortality in the overall ARDS patient population. Exome-seq is a powerful tool to identify potential new biomarkers for ARDS. We selectively validated three SNPs which have not been previously associated with ARDS and represent potential new genetic biomarkers for ARDS. Additional validation in larger patient populations and further exploration of underlying molecular mechanisms are warranted.

## Introduction

Acute respiratory distress syndrome (ARDS), a severe form of acute lung injury, is characterized by the inflammation and fluid build-up in the alveoli of the lungs, which reduces the ability of oxygen to cross over into the blood stream [1], [2]. ARDS has an extremely high mortality rate where over a third of sufferers die, and many of the survivors experience complications such as brain damage due to prolonged oxygen deprivation [3], [4]. The mortality rate is even higher in cases with common comorbidities such as sepsis with suspected pulmonary source (40.6%) and witnessed aspiration (43.6%) [3]. ARDS is estimated to have an age-adjusted incidence of 86.2 new cases per 100,000 person-years in adults age 15 and older. The total number of cases estimated to occur yearly in the US is about 190,000 [3]. Pneumonia and sepsis are most common causes of ARDS, and Sepsis is the leading cause of ARDS. There is a paucity of effective and specific therapy to ARDS though low tidal volume ventilation has been demonstrated for some therapeutic utilities [5]–[8]. This is because the etiology and pathology of the disease are still not well understood and there remains a need for new specific and effective preventative measures and treatments.

The role of genetics in ARDS is increasingly recognized and it has recently been shown that complex diseases can be between 50 and 90% genetically determined [9]. Biomarkers that have been previously studied that are present in blood serum include surfactant-associated proteins (SP-A, B, and C), Mucin-associated antigens (KL-6 and MUC1), Cytokines (IL-1, 2, 6, 8, 10, and 15, TNFα), endothelium activation markers (E-selectin, L-selectin, I-CAM-1, V-CAM-1, and VWF), and neutrophil activation markers (MMP-9, LTB4, and Ferritin). Cytokine levels have been identified as a moderately effective measure of severity [10]. Additional biomarkers of ARDS severity have been obtained from breath analysis, including hydrogen peroxide levels and breath acidity [11]. Pre-B cell Colony Enhancing Factor (PBEF), also called nicotinamide phosphoribosyltransferase (NAMPT), was identified previously as a novel biomarker of ARDS by our group [12]. Analysis of two SNPs in the human PBEF promoter revealed an association with ARDS. The −1535T variant allele was associated with a decreased susceptibility to ALI/ARDS and a better outcome in septic patients in a Caucasian population when compared with patients without the variation. The −1001G variant allele was associated with increased susceptibility to acute lung injury and ARDS in African American and Caucasian populations. The −1001G variant was also associated with a higher ICU mortality rate in septic patients in a Caucasian population [6], [13]–[15]. Despite the previous identification of several available biomarkers, their available data are inconsistent and clinical relevance has not yet been established.

With the development of next-generation sequencing technologies and improvements in data analysis capabilities, it is now feasible to sequence and analyze whole genomes within a couple of days [16]. However, the cost of whole genome sequencing is still a prohibitive factor for sequencing more samples. Whole exome sequencing (WES) is faster and less expensive than whole-genome sequencing, making it ideal for the study of variants that cause changes to the protein-coding regions of genes [17]. WES has been used to identify genetic risk factors for both Mendelian and complex diseases alike [17]–[19]. The purpose of this study was to discover new biomarkers for ARDS using WES. Exome sequencing of 96 ARDS patient DNA samples from the ARDSnet (www.ardsnet.org) and 48 race, gender and age matched normal healthy control subject DNAs from Coriell (www.coriell.org) was performed using Illumina's HiScanSQ system. By comparing SNP analysis of whole exome sequence data between the ARDS patient population (96 patients) and the normal healthy controls from Coriell (48 subjects) as well as the 1000 Genomes Project (440 total, 379 European Ancestry (EUR), 61 African Americans in the southwest (ASW), www.1000genomes.org) [20], we have identified a number of coding SNPs potentially associated with ARDS susceptibility. We also performed regression analyses within the ARDS patient population to assess association of some newly identified SNPs with ARDS severity (APACHE II score) and outcome (60 day mortality). In addition, we validated three SNPs (rs78142040, rs9605146 and rs3848719) in an additional 117 ARDS patients for a total of 213 cases using TaqMan genotyping assays (Life Technologies) to substantiate their associations with the susceptibility, severity and outcome of ARDS.

## Materials and Methods

### ARDS patients and healthy control subjects

To perform this case-control study, we used 213 ARDS patient DNA samples from the ARDSnet (www.ardsnet.org) and 440 healthy control subjects (379 EUR and 61 ASW) from the 1000 Genomes Project (www.1000genomes.org). The African Ancestry 1000 Genomes Project panel used in our study is ASW (Americans of African Ancestry in Southwest USA). The European Ancestry 1000 Genomes Project panels used in our study include CEU (Utah residents with Northern and Western European ancestry), FIN (Finnish in Finland), GBR (British in England and Scotland), IBS (Iberian population in Spain), and TSI (Toscani in Italia). Clinical information for 213 ARDS cases was obtained from the NHLBI ARDS network 05: Fluid and Catheter Treatment Trial [21], [22] as managed by the Biologic Specimen and Data Repository Information Coordinating Center (BioLINCC, http://biolincc.nhlbi.nih.gov.home). Limited demographic variables for normal control subjects were obtained from the 1000 Genomes Project (www.1000genomes.org).

### Exome-seq and data analysis

Exome sequencing was performed on 96 ARDS cases using HiScanSQ (Illumina, CA, USA). Briefly, the libraries for exome sequencing were created using the TrueSeq Exome Enrichment Kit (http://www.illumina.com). Paired-end sequencing with 101 base pair read lengths was performed using Illumina’s HiScanSQ, which provides a minimum average coverage depth of 50×. Consensus Assessment of Sequence And VAriations (CASAVA) software was used for the conversion of HiScanSQ reported.bcl files to.fastq format and for demultiplexing (http://www.illumina.com). The sequences were aligned to the hg19 human reference genome and variants alleles were called using the Genome Analysis Toolkit (GATK) (http://www.broadinstitute.org/gatk/). Sequencing data are submitted to the NCBI BioProject database (Accession ID: 262819, http://www.ncbi.nlm.nih.gov/bioproject/262819).

Both the lab-generated data for ARDS patient samples and the 1000 Genomes Project controls were processed using the GATK methodology [23]. GATK was also used to generate the list of SNPs from the sequence data. Sequencing data of the patient samples were considered to be high-confidence if the Phred-like quality was a minimum of 20 and there were at least 4× coverage depths. SNPs from the two data sources were merged by location from alpha ordered datasets and minor allele counts were determined from the merged data.

For the analysis of candidate SNPs associated with the ARDS susceptibility, the ARDS SNP data were compared with the 1000 Genomes Project SNP data. The total control sample size in the 1000 Genomes Project is 1092, and the data identifies about 15 million SNPs which underwent stringent quality control [20], [24]. We studied the 440 ASW and EUR samples obtained from this dataset. In racially stratified analyses, the ASW population in the 1000 Genomes Project was used as a control population for the African American ARDS samples and the EUR population was used as a control for the Caucasian ARDS population (Table S1). SNPs in HWE in the cases with high data quality were selected for genotype validation. The detailed association analysis and Hardy-Weinberg equilibrium analysis are provided in Supporting Information S1 [25], [26].

For the analysis of candidate SNPs associated with the ARDS severity, the correlation between SNPs and the APACHEII score (a measure of the severity of a disease in adult patients) as well as the number of ventilator-free days per 28 days (an indication of a patient’s ability to breathe on their own) was conducted.

For the identification of candidate SNPs associated with the ARDS outcome, the logistic regression analysis between SNPs and 60 day mortality was performed.

Following the results of the genetic association study the data were further analyzed through the use of the Variant Analysis component of SNP & Variation Suite v8.2.0 (SVS v8.2.0, Golden Helix, Inc., Bozeman, MT, www.goldenhelix.com). This software ranked the SNPs in order of likely importance based on location, as well as amino acid change predictions. This information was joined with predictions of protein functional effect changes made by Sift and Provean (http://provean.jcvi.org/index.php) as well as Polyphen2 (http://genetics.bwh.harvard.edu/pph2/index.shtml). Ingenuity Pathway Analysis Software (www.ingenuity.com/) from Ingenuity Systems was used to screen for SNPs which are likely to alter the function of relevant biologic pathways. To accomplish this a list of the genes that contain SNPs with χ^2^ p-value of<2.95×10^−7^ in at least 2 of the 5 main susceptibility comparisons and a χ^2^ p-value of <0.01 in all of the 5 main comparisons of exome sequence data (All ARDS vs. 1000 Genomes, All Sepsis vs. 1000 Genomes, All Pneumonia vs. 1000 Genomes, African American ARDS vs. ASW1000 Genomes, and Caucasian ARDS vs. EUR 1000 Genomes) was submitted to IPA. Additional information on the statistical methods preformed in this study can be found in Supporting Information S1, which contains descriptions of the principal component analysis and genomic inflation factor calculations [27]–[31].

### Genotyping of Selected Candidate SNPs

Three selected SNPs (rs78142040; rs9605146, and rs3848719) in the additional 117 ARDS patient DNA samples from the NHLBI Ardent were genotyped using TaqMan human SNP genotyping assays on the ViiA 7 Real Time PCR System (Life Technologies, Grand Island, NY) according to the supplier’s instruction. Genotyping accuracy was validated using 10 previously exome-sequenced samples in-lab. Genotyping data from the additional samples was combined with the existing 96 patient sample to increase sample size for the 3 SNPs and association tests were repeated using the total 213 patient population.

## Results

### Identification of novel coding SNPs associated with the ARDS susceptibility

**Figure 1:**
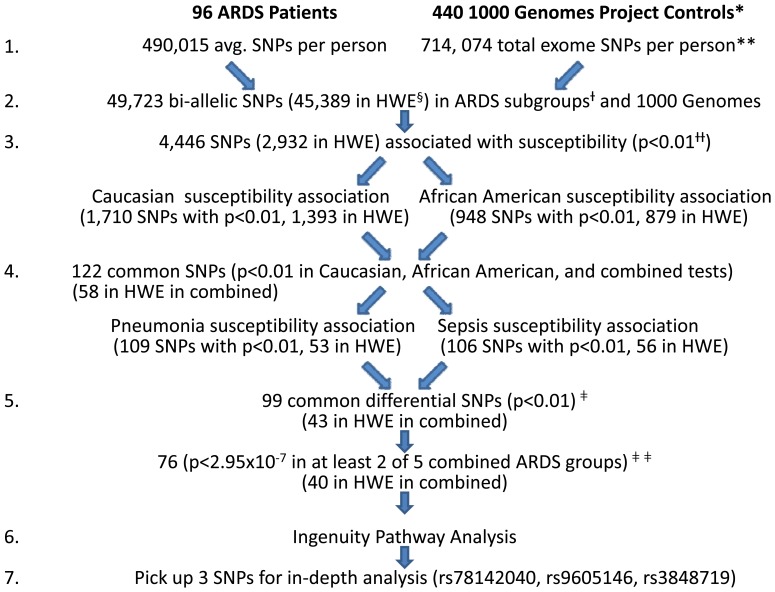
Pipeline of the exome-seq data analysis workflow. After processing the data using the GATK pipeline, this filtering workflow was derived to identify SNPs which were associated with measures of susceptibility across the racial and etiology groups of cases. SNPs were filtered based on strength of association, coding effect, and functional prediction prior to testing for association with other ARDS phenotypes. *, The sample contains African American and Caucasian patients, so the EUR and ASW healthy controls from 1000 Genomes were used for comparison; **, In the 1000 Genomes Project exome sequence, the same 714,074 SNPs are present for all 440 EUR and ASW; §, HWE = Hardy Weinberg Equilibrium, p>0.0001; +, African American with pneumonia, African American with sepsis, Caucasian with pneumonia, Caucasian with sepsis; + +, χ^2^ test of ARDS vs. respective 1000 Genomes Project control groups; ‡, SNPs with P-value <0.01 in the overall comparison, Caucasian ARDS comparison, and African American comparison with 1000 Genomes were filtered further by p<0.01 in the sepsis comparison and pneumonia comparison; ‡ ‡, All ARDS cases, all pneumonia cases, all sepsis cases, all African American cases, all Caucasian cases.

**Figure 2:**
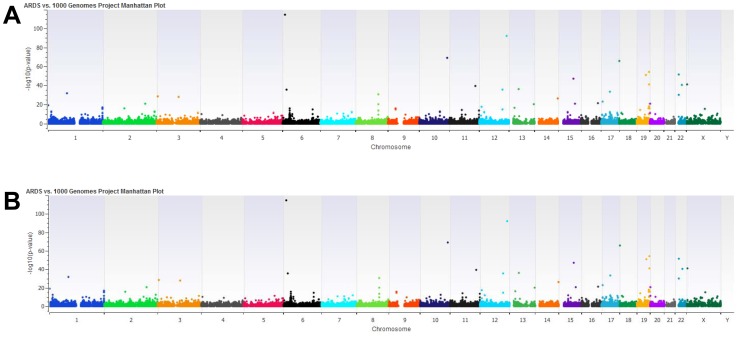
Manhattan plot of ARDS patients and 1000 genomes project controls. A number of strong associations with susceptibility to ARDS were observed using a χ^2^ test. (**A**) A Manhattan plot of the whole exome sequence all ARDS cases vs. European Ancestry and ASW 1000 Genomes Project controls created using SVS v8.2.0. This is a graphic representation of the chromosome location (x axis) vs. the –log10 (χ^2^ p-value) of the allele frequencies. SNPs whose chi-square tests yield a smaller p-value fall higher on the log scale are more significant [42]. (**B**) The same Manhattan plot with a zoomed Y-axis.

In order to identify novel coding SNPs associated with the ARDS susceptibility, we performed the exome-seq of 96 ARDS patient DNAs. These patients consisted of 70 Caucasian and 26 African Americans (Table 1). In Caucasian patients, 37 cases were due to the initiating etiology of sepsis and 33 were due to pneumonia. In African American patients, 11 cases were due to the initiating etiology of sepsis and 15 were due to pneumonia. We detected 1,382,399 SNPs in 96 ARDS patients by exome-seq (Table 2) and 490,015 SNPs per person on average (Figure 1). Among them, 169,376 SNPs matched records from 625 healthy control subjects in the 1000 human genome project. From 169,376 SNPs, there are 49,723 bi-allelic SNPs out of 50,190 total SNPs in all ARDS patient subgroups based on race and initiating etiologies: Caucasian sepsis, Caucasian pneumonia, African-American sepsis and African American pneumonia. Of our 1,382,399 ARDS SNPs, 608,723 were common between the sepsis and pneumonia cases while 369,639 and 404,037 are non-overlapping SNPs, respectively. There are 442,235 common SNPs between our African American cases and Caucasian cases while 337,738 and 602,426 are non-overlapping SNPs, respectively. 87.8% of the 1,382,399 ARDS SNPs (i.e., 1,213,023 ARDS SNPs) are not found in the 1000 Genomes Project Exome, but 85.4% of the 1,213,023 ARDS SNPs (i.e., 1,035,921 SNPs) were assigned RS numbers, suggesting our data collection and processing are reliable. By comparing the frequencies of the minor alleles in those newly detected SNPs in 96 ARDS patients with those in 440 Caucasian and African-Americans from the Southwest healthy control subjects of the 1000 human genome project, we found that there are 3,867 differential SNPs (p<0.01) (Figure 2). In Caucasians, between ARDS patients and healthy controls, there are 788 differential SNPs (p<0.01). In African-Americans, between ARDS patients and healthy controls, there are 948 differential SNPs (p<0.01). There are 122 common differential SNPs (p<0.01) between either Caucasian or African American patients or healthy controls. When we examined sepsis- or pneumonia-initiated ARDS separately, we found that 106 and 109 differential SNPs (p<0.01), respectively. Between them, there are 99 common differential SNPs (p<0.01). When the Bonferroni correction (p<2.95×10^−7^) was applied, 76SNPs remains significantly different. These SNPs are potentially novel coding SNPs associated with the ARDS susceptibility.

**Table 1:** Participant demographics and comorbidities for the ARDS cases.

Whole-exome SequencingSample group	Participants*	Gender(%male)	Age± SD	APACHEIIscore ±SD	Ventilator-free daysper 28 days ±SD	60-day mortality(% dead at 60 days post-onset)
ARDS total	96	46.9	50±14.6	97.2±30.4	12.6±10.2	29.4
Caucasian ARDS	70	44.3	50.7±13.8	94.5±30.2	13.6±10.0	23.9
African American ARDS	26	53.8	47.8±16.6	104.6±30.2	9.9±10.5	44
ARDS with Sepsis total	48	50	52.0±15.1	105.2±32.2	10.2±10.1	38.3
Caucasian ARDS with Sepsis	37	43.2	51.8±15.0	102.6±31.1	10.5±9.8	33.3
African American ARDS with Sepsis	11	72.7	52.9±16.1	113.6±35.8	9.3±11.7	54.5
ARDS with Pneumonia total	48	43.8	47.9±13.9	88.9±26.2	14.9±9.87	20
Caucasian ARDS with Pneumonia	33	45.5	49.5±12.4	85.0±26.6	17.0±9.2	12.9
African American ARDS with Pneumonia	15	40	44.1±16.6	97.6±24.0	10.3±10.0	35.7
**TaqMan Genotyping** **Sample group**	**Participants**	**Gender** **(%male)**	**Age± SD**	**APACHEII** **score ±SD**	**Ventilator-free days** **per 28 days ±SD**	**60-day mortality** **(% dead at 60 days post-onset)**
ARDS Total	117	46.2	50.8±16.6	105.1±32.7	12.0±10.5	31
Caucasian ARDs	75	40	52.1±15.4	104.3±31.4	11.8±10.5	29.2
African American ARDS	17	64.7	51.6±22.8	113.3±33.8	12.2±10.6	29.4
Other ancestry ARDS	25	52	46.4±15.3	101.8±36.2	12.6±10.7	37.5
ARDS with Sepsis total	59	52.5	51.5±17.3	112.9±31.5	11.3±11.0	37.9
Caucasian ARDS with Sepsis	34	50	51.1±15.6	113.6±29.7	10.9±11.1	33.3
African American ARDS with Sepsis	9	66.7	56.6±27.1	121.8±33.0	12.9±11.5	33.3
Other ancestry ARDS with Sepsis	16	50	49.6±14.8	106.4±34.7	11.4±11.0	50
ARDS with Pneumonia total	58	39.7	50.1±16.0	96.9±32.3	12.7±9.9	23.6
Caucasian ARDS with Pneumonia	41	31.7	52.9±15.3	96.4±31.0	12.6±10.0	25.6
African American ARDS with Pneumonia	8	62.5	46±16.9	103.8±34.3	11.4±10.2	25
Other ancestry ARDS with Pneumonia	9	55.6	46.8±15.2	92.4±39.7	14.9±10.3	12.5
**Total ARDS**	**Participants**	**Gender (%male)**	**Age± SD**	**APACHEII score ±SD**	**Ventilator-free days per 28 days ±SD**	**60-day mortality (% dead at 60 days post-onset)**
All ARDS patients	213	46.5	50.4±15.7	101.6±31.9	12.3±10.3	30.2

*8 samples, 4 exome sequenced and 4 TaqMan genotyped ARDS patients did not have these phenotypes available. An additional 2 exome sequenced ARDS patients did not have ventilator-free day’s data. In addition to the 8 patients missing severity and mortality phenotype data, 2 patients were excluded from the regression because their phenotypes were thought to be missing until after the regressions were completed.

**Table 2:** Summary of the filtering applied to candidate SNPs.

Criteria for filtering	Number of remaining variants
SNPs detected in ARDS SNPs	1,382,399
matched in 1000 Genomes Project	169,376
common across 4 ARDS race and etiology groups*	50,190
?^2^ p-value <0.01 in top 5 ARDS groups**	99
?^2^ p-value <2.95×10^−7^ in at least 2/5 top 5 ARDS groups	76(in 65 genes)
coding variants	38(in 32 genes)
nonsynonymous	20
synonymous variants	18

*African American with pneumonia, African American with sepsis, Caucasian with pneumonia, Caucasian with sepsis subgroups. Out of the 50,190 SNPs, 49,723 are bi-allelic. **, All ARDS, all pneumonia, all sepsis, all African American, all Caucasian groups.

### Pathway Analysis

These 76 SNPs occur in 65 genes. To determine the functional consequences of these SNPs, Ingenuity Pathway Analysis was conducted on these 65 genes to identify biologic pathways in which these genes function. The top canonical pathway is *graft-versus-host disease signaling* and included 6 genes that contained associated SNPs, comprising 13% of the genes involved in the pathway (p = 8.07×10^−9^). The top 5 canonical pathways additionally include *autoimmune thyroid disease signaling*, *Nur77 signaling in T lymphocytes*, *calcium-induced T lymphocyte apoptosis*, and *B-cell development signaling* (Table 3). Among the 76 remaining significantly different SNPs after the Boneferroni correction (Figure 1), 38 SNPs are coding variants. Of these, 20 SNPs can cause nonsynonymous amino acid changes while 18 SNPs can cause synonymous amino acid changes (Table 2).

**Table 3:** Pathway analysis.

Top Canonical Pathways	p-value*	Ratio**	Molecules
Graft-versus-Host Disease Signaling	8.07×10^−9^	6/48	KIR2DL1/KIR2DL3, HLA-DRB1, HLA-B, HLA-DQA1, HLA-DQB1, HLA-DRB5
Autoimmune Thyroid Disease Signaling	4.35×10^−7^	5/49	HLA-DRB1, HLA-B, HLA-DQA1, HLA-DQB1, HLA-DRB5
Nur77 Signaling in T Lymphocytes	9.37×10^−7^	5/57	HLA-DRB1, HLA-DQA1, HLA-DQB1, HLA-DRB5, SIN3A
Calcium-induced T Lymphocyte Apoptosis	1.68×10^−6^	5/64	HLA-DRB1, HLA-DQA1, HLA-DQB1, ITPR1, HLA-DRB5
B Cell Development	3.75×10^−6^	4/34	HLA-DRB1, HLA-DQA1, HLA-DQB1, HLA-DRB5

Top canonical pathways as predicted from the 65genes containing the76 SNPs that were identified using χ^2^tests. Pathway predictions were done using the Core Analysis function of Ingenuity Pathway Analysis. *, P-Value of <0.05 indicates a non-random association between the genes and pathway; **, Ratio of the number of genes in the dataset involved in the pathway to the total number of genes in the pathway.

### Selected validations of three SNPs (rs78142040, rs9605146 and rs3848719)in additional 117 ARDS patients

To validate the result of SNP identification by exome-seq, we selectively genotyped three SNPs (rs78142040, rs9605146 and rs3848719) in an additional 117 ARDS patients using the TaqMan genotype assay (Table 4). We then examined their association with the susceptibility, severity and outcome to ARDS in a combined 213 ARDS patients (96 by exome-seq +117 by TaqMan = 213) (Table 5, Table S2, Table S3, and Table S4).

**Table 4:** Profile of 3 SNPs.

	rs3848719	rs9605146	rs78142040
Location	20∶44596545	22∶17265194	X:2832771
Gene ID	ZNF335	XKR3	ARSD
Call Rate (Cases+Controls)	0.962	0.995	0.991
Call Rate (Cases)	0.883	0.986	0.972
HWE P-value (Cases)	2.86E-4	0.422	1.18E-3
HWE P-value (Controls)	0.887	3.72E-5	1
HWE P-value (Cases+Controls)	0.034	1.94E-12	0.471
Reference: Alternate Allele	G>A	G>A	C>T
Alt. Allele Freq. (Cases)	39.4%	38.6%	22.0%
Alt. Allele Freq. (Controls)	38.5%	4.0%	0%
Number AA (%) (Cases)	41 (21.8%)	34 (16.2%)	2 (1.0%)
Number AA (%) (Controls)	66(15.0%)	4(0.9%)	0(0%)
Number Ar (%) (Cases)	66 (35.1%)	94 (44.8%)	87 (42.0%)
Number Ar (%) (Controls)	207 (47.0%)	27 (6.1%)	0 (0%)
Number rr (%) (Cases)	81 (45.1%)	82 (39.1%)	118 (57.0%)
Number rr (%) (Controls)	339 (77.0%)	35 (8.0%)	0 (0%)
SNP classification	Coding	Coding	Intronic
Coding classification	Synonymous	Nonsynonymous	Intronic
Reference amino acid	S	P	NK
Alternate amino acid	S	L	NK
Provean prediction (cutoff = −25)^1^	Neutral	Deleterious	NK
Sift prediction (cutoff = 0.05)^1^	Tolerated	Tolerated	NK
Pph2 prediction^2^	NK	benign	NK

NK: Not known, HWE, Hardy-Weinberg equilibrium; AA, alternate genotype or homozygous minor genotype; Ar, heterozygous genotype; rr, reference genotype or homozygous major genotype; 1, http://provean.jcvi.org/genome_submit.php; 2, http://genetics.bwh.harvard.edu/pph2/.

**Table 5:** Overall association summary.

	rs3848719	rs9605146	rs78142040
Susceptibility (cases vs. controls)			
Chi-squared p-value	0.780	1.68E-59	3.64E-47
Odds Ratio (95% CI)^1^	1.04(0.81–1.33)	15.16(10.25–22.41)	498.09 (30.83–8,047.51)
Severity (ventilator-free days/28 days)			
p-value	NS	NS	NS
Odds Ratio (95% CI)^2^	NS	NS	NS
Severity (APACHE II score)			
p-value	0.032	NS	0.061**
Odds Ratio (95% CI)^2^	0.55 (0.31–0.96)	NS	2.60(0.93–7.26)
Outcome (60-day mortality)			
p-value	NS*	NS	0.017
Odds Ratio (95% CI)^2^	NS	NS	2.04 (1.13–3.68)

NS, Not significant; * significantly associated in genotyped Caucasian, pneumonia, and Caucasian pneumonia subgroups; **, for 117 genotyped samples only, not in total 213; 1, Odds ratio for alternate allele (allelic test); 2, additive genotypic model.

rs78142040 has a major allele C and a minor allele T and is found on the X chromosome position X: 2832771 in the Arylsulfatase D gene (ARSD). The SNP was determined to lie within a histone mark of intron 6 using the UCSC Genome Browser (http://genome.ucsc.edu/) and could potentially play a role in regulation of expression. The ARSD was associated with bone and cartilage development and was identified previously as having involvement in sphingolipid metabolism and as a potential biomarker for chronic lymphocytic leukemia [32]. The SNP is in Hardy-Weinberg Equilibrium (HWE p>1×10^−4^) in the 1000 Genomes controls and the ARDS population and subgroups.

The SNP is associated significantly with susceptibility (p<2.95×10∧−7) in the total 213 patient population (MAF = 0.22) and the subgroups when compared with those from the 1000 Genomes Project (MAF = 0.00) (Table 5, Additional file 6: Table S5). rs78142040 approaches association with APACHEII score when the score quartiles are compared for the genotyped ARDS patients (p = 0.061, OR = 2.603, 95% CI = 0.933–7.260) (Table 5, Table S4). rs78142040 is associated with the 60-day mortality in the total ARDS population (p = 0.017, OR = 2.039, 95%CI 1.130–3.681) (Table S3).

rs9605146 (also known as rs114989947) has a major allele G and minor allele A. It is a nonsynonymous SNP found within exon 4 of chromosome 22 (22∶17265194) in the “XK, Kell blood group complex subunit-related family, member 3” gene (XKR3) and causes a predicted amino acid change from proline to leucine. The protein encoded by XKR3 is a homolog of XK, which is a putative membrane transporter [33]. XKR3 has not been previously associated with human disease. This amino acid change has a deleterious effect predicted by a Provean score of −5.494, where a score of maximum −2.5 is considered to be deleterious. The SNP is in HWE (p>1×10^−4^) in the EUR 1000 Genomes Project samples as well as in the ARDS population and subgroups. rs9605146 is associated with disease susceptibility (p<2.95×10^−7^) in the total ARDS population (MAF = 0.39) and subgroups when compared with the 1000 Genomes controls (MAF = 0.04) with the exception of the African Americans when the sepsis and pneumonia etiologies are analyzed individually (Table 5, Table S6). The SNP also approaches significant association with 60-day mortality in the exome-sequenced patients with pneumonia (p = 0.080).

rs3848719 has a major allele of G and a minor allele A. It is a synonymous SNP in the 5^th^ exon of the Zinc-Finger/Leucine-Zipper Co-Transducer NIF1 gene in chromosome 20 (ZNF335, location 20∶44596545). The ZNF335 gene is expected to play a role in transcription regulation and is involved in neural progenitor cell proliferation and self-renewal. It is associated with the disease microcephaly [34], [35]. The SNP is in Hardy-Weinberg Equilibrium (HWE p>1×10^−4^) in the 1000 Genomes controls and the ARDS population and subgroups.

The SNP was not associated with susceptibility in the total ARDS population (MAF = 0.39) when compared with the 1000 Genomes controls (MAF = 0.385) (Table 5, Table S7), however rs3848719 is associated with APACHEII score when the score quartiles are compared for total ARDS patients (p = 0.032, OR = 0.549, 95%CI = 0.313–0.96). The SNP is associated with 60-day mortality in the TaqMan genotyped Caucasian ARDS (p = 0.012, OR = 2.753, 95% CI = 1.196–6.336), TaqMan genotyped pneumonia (p = 0.032, OR = 2.511, 95% CI = 1.053–5.984), and TaqMan genotyped Caucasians with pneumonia (p = 0.012, OR = 4.045, 95% CI = 1.219–13.433).

## Discussion

### Overview of WES Findings

Whole-exome sequencing (WES) had been performed in 96 ARDS patients from the ARDSnet with the intent of identifying coding SNPs whose minor allele frequencies are significantly different in ARDS than those of healthy controls and of identifying those novel SNPs who may be predictors of ARDS severity and outcome. In the overall ARDS population 1,382,399 SNPs were detected by exome-seq (Table 2) and 490,015 SNPs per person on average (Figure 1) compared to 714,074 SNPs per person from 625 healthy control subjects in the 1000 Genome Project. Among them, only 169,376 SNPs overlapped between two populations. The majority of un-overlapped SNPs in ARDS patients may represent ARDS specific SNPs barring the individual variability, sequencing error and data analysis discrepancy. From 169,376 SNPs, there are 49,789 bi-allelic SNPs in all ARDS patient subgroups based on race and initiating etiologies: Caucasian sepsis, Caucasian pneumonia, African-American sepsis and African American pneumonia. These SNPs may represent sepsis or pneumonia etiology specific SNPs of ARDS. The reason why we initially focused on the identification of novel coding SNPs associated with ARDS in sepsis and pneumonia origins was that in the original ARDS patient population, sepsis and pneumonia etiologies accounted for most cases [21], [22]. We selectively genotyped and validated three SNPs (rs78142040, rs9605146 and rs3848719) in an additional 117 ARDS patients using the TaqMan genotype assay and performed in depth association analyses of these SNPs with the susceptibility, severity and outcome to ARDS in a combined 213 ARDS patients (96 by exome-seq +117 by TaqMan = 213). These validations lend a solid support to the validity and prowess of novel ARDS associated SNP identifications by exome-seq. This study provides a rich resource for further experimentation and replication to develop and establish new genetic biomarkers and therapeutic targets to ARDS.

### Validation of selected three SNPs

Among three selectively validated SNPs (rs78142040, rs9605146 and rs3848719) in an additional 117 ARDS patients, rs78142040 in the ARSD gene is associated with increased ARDS susceptibility in the overall ARDS population (213 patients) as well as all racial and comorbidity subpopulations. It approaches significant association with an increase in APACHEII score (p = 0.061) when samples in the highest and lowest score quartiles are compared in ARDS patients. rs78142040 is associated significantly (p<0.05) with an increase in 60-day mortality in the total ARDS population (p = 0.017, OR = 2.039, 95%CI 1.130–3.681). The molecular mechanisms underlying these associations are presently unknown. The ARSD gene encodes a sulfatase that is associated with bone and cartilage development and has been identified previously as having involvement in sphingolipid metabolism (involved in signal transmission and cellular recognition) and as a potential biomarker for chronic lymphocytic leukemia [32]. ARSD protein isoforms have a highly conserved catalytic peptide domain when compared with other arylsulfatases [36], [37]. ARSD is widely expressed and is suspected to play a role in housekeeping or multiple other processes, however specific substrates have not been identified [38]. It was reported that there were changes in activities of lung liposomal enzymes including sulfates during ARDS [39]. It may be interesting to explore whether rs78142040 causes the differential expression of the ARSD gene, thus sulfatase activity, which may link its role in the pathogenesis of ARDS.

rs9605146 in the XKR3 gene is associated with increased susceptibility in the ARDS population and all subgroups except the African American with sepsis etiology group and the African American with pneumonia etiology group when analyzed individually. XKR3 is a member of the XK/Kell complex in the Kell blood group system. XKR3 is a homolog of XK, which is a putative membrane transporter. XK is associated with McLeod syndrome (characterized by late-onset abnormalities in the central nervous system and neuromuscular system) and red cell acanthocytosis [33]. XKR3 has previously been indicated as a potential biomarker for blood transfusion compatibility [40]. While it is currently unknown what underlies the association of rs9605146 with susceptibility in ARDS, it causes a deleterious amino acid coding change from proline to leucine in the XKR3 gene as predicted by Provean (score = −5.494). These observations make rs9605146 a legitimate candidate for further study of its role in the pathogenesis of ARDS.

rs3848719 in the ZNF335 gene is not associated significantly with susceptibility, however the SNP is associated with a decreased APACHEII score when the highest and lowest score quartiles are compared in the total ARDS population (p = 0.032, OR = 0.55, 95%CI 1.27–2.05), and with an increased 60-day mortality in Caucasian and pneumonia groups (p<0.05). It is a synonymous SNP in the 5^th^ exon of the Zinc-Finger/Leucine-Zipper Co-Transducer NIF1 gene (ZNF335). ZNF335 gene is involved in neural progenitor cell proliferation and self-renewal as a component of the vertebrate-specific, trithorax H3K4-methylation complex. ZNF335 is associated with the disease microcephaly (a neurodevelopmental disorder), small somatic size and neonatal death. The gene is essential as homozygous knockout mouse models have a lethal effect [34], [35]. The role of the gene in cellular differentiation and gene expression could implicate an effect on the fundamental physiology and neural signaling in the lungs, contributing to the pathogenesis of ARDS.

### Limitations

**Figure 3:**
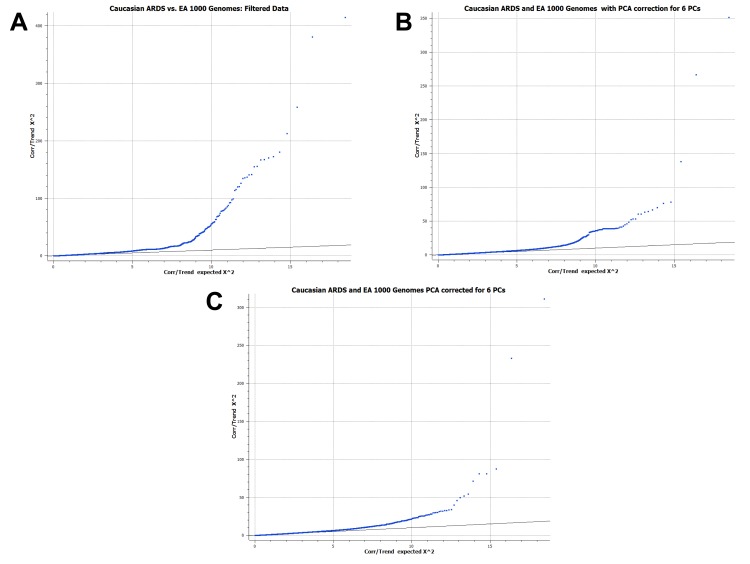
Quantile-quantile plots of Caucasian ARDS and EUR 1000 genomes. In the example of our Caucasian cases and EUR controls, we observe that correction for principal components improves the fit of our data with the expected distribution. (**A**) QQ plot of expected χ^2^values versus the actual χ^2^values for the genotypic trend test of case-control status. The data are filtered on HWE, LD, and SNP call rate but not PCA corrected. (**B**) QQ plot of expected χ^2^values versus the actual χ^2^values for the genotypic trend test of case-control status. The data have been filtered and corrected for 6 PCs. (**C**) QQ plot of expected χ^2^values versus the actual χ^2^values for the genotypic trend test of case-control status. The data have been filtered and corrected for 6 PCs and undergone sample outlier removal.

Although we applied the Bonferroni correction (p<2.95×10^−7^) and several SNP filtering steps during our data analysis as well as validations of three selected candidate SNPs to ARDS, our data come with potential limitations. First, we only performed exome-seq of 96 ARDS samples. Although we would argue that this is a very reasonable sample size considering the restriction of high exome-seq cost per sample, even though the exome-seq cost per sample is cheaper than whole genome-seq per sample, the sample size is not large. Our 76 SNPs which are associated strongly with susceptibility are all present in an age and race matched 48 sample control set, which will be used to validate our findings in further studies (117,35 out of the 169,376 SNPs which are in the ARDS cases and 1000 Genomes Project are found in this control set). Confirmation of our findings in larger patient populations is warranted. Second, during analysis of SNP associations with ARDS susceptibility, we used the healthy control subjects from the 1000 Genome Project. Both ARDS patients and healthy control subjects do not derive from the same population. Since population admixture is assumed in the African American cases, we have elected to compare these cases with the ASW subset of the 1000 Genomes Project African Ancestry panel. We feel this is the best fitting control group due to the observed reduction in genomic inflation factor (inflation factor = 1.18 when compared with ASW, after filtering for informative markers based on HWE, call rate, number of alleles, and LD) compared to the total African Ancestry controls (inflation factor = 1.70), YRI alone (inflation factor = 1.59), or LWK alone (inflation factor = 1.98) [41]. An ancestry-informative SNP panel with good coverage of our dataset was not available. Although we have applied HWE, PCA analysis and Q–Q plot determination as well as race specific comparison to filter the identified SNPs, it may not totally correct the population admixtures (Figure 3, Figure S1, Figure S2, Table S8). Two of the SNPs (rs9605146, control MAF 4.0% and rs78142040, control MAF 0% respectively) have extremely minor allele frequencies which causes inflation of the type-1 error of the Goodness-of-fit test for HWE [26]. In this study, we explicitly searched for SNPs in which the MAF differed between cases and controls, so we expect that we might see some deviation where the minor alleles are rare in healthy controls. The observed associations with other disease phenotypes within our case cohort support our conclusion that variations at these loci contribute to disease. Replication of our findings in larger and different populations may strengthen and develop the candidate SNPs identified here as true genetic biomarkers of ARDS.

### Conclusion

The primary focus of this study was to identify new and novel SNPs associated with ARDS susceptibility, severity and outcome using whole exome-sequencing. We have identified a number of potential ARDS associated SNPs, which has demonstrated that WES is a powerful tool to identify new biomarkers in ARDS. We selectively validated 3 SNPs that are associated with the susceptibility (rs78142040 and rs9605146), severity (rs3848719) and outcome (rs78142040 and rs3848719) of ARDS. More validations in larger and different patient populations as well as further investigation of the underlying molecular mechanisms are warranted to establish them as true new diagnostic and therapeutic targets for ARDS.

## Supporting Information

Figure S1 **The scree plots of the eigenvalues generated by principal component analysis.** The largest eigenvalues are used in corrections for population structure. (**A**) The All ARDS and ASW+EUR 1000 Genomes population eigenvalues. 535 principal components were measured and the largest eigenvalue is 7.32. (**B**) The Caucasian ARDS and EUR ARDS population eigenvalues. 449 principal components were measured and the largest eigenvalue is 1.52. (**C**) The African American ARDS and ASW 1000 Genomes population eigenvalues. 86 principal components were measured and the largest eigenvalue is 0.94.
(DOCX)

Figure S2 **The quantile-quantile plots of genotypic trend test χ^2^ values for the African American ARDS and ASW 1000 Genomes population were derived using SVS v8.2.0.** The straight line on each plot represents y = x. (**A**) QQ plot of expected χ^2^values versus the actual χ^2^values for the genotypic trend test of case-control status. The data are filtered on HWE, LD, and SNP call rate but not PCA corrected. (**B**) QQ plot of expected χ^2^values versus the actual χ^2^values for the genotypic trend test of case-control status. The data are corrected for the 2 largest principal components. (**C**) QQ plot of expected χ^2^values versus the actual χ^2^for the genotypic trend test of case-control status. The data have been filtered and corrected for 2 PCs and undergone sample outlier removal.
(DOCX)

Table S1 **A summary of the comparison groups used for the genetic association analysis.** Association of the exome-seq SNPs with susceptibility was explored by comparing 96 ARDS patients to 440 controls from the 1000 Genomes Project. Analysis was stratified by race and etiology.
(DOCX)

Table S2 **A summary of the susceptibility χ^2^ tests for the 3 SNPs.** The allelic chi-square test p-values for the exome sequenced ARDS patients ARDS patients and subgroups compared with the 1000 Genomes Project participants and subgroups, TaqMan genotyped ARDS patients and subgroups compared with the 1000 Genomes Project participants and subgroups, and the total ARDS patient population and subgroups compared with the 1000 Genomes Project participants and subgroups. P-values were considered to be significant if they were smaller than the Bonferroni corrected p-value of 2.95×10^−7^.
(DOCX)

Table S3 **Logistic regression with 60-day mortality from the day of diagnosis was used to assess SNP association with outcome.** Included in this table are the p-values of the logistic regression of 60-day mortality against genotype using an additive model in the ARDS exome samples, TaqMan genotyped samples, and total ARDS samples. Associations were considered significant if p<0.05.
(DOCX)

Table S4 **Logistic regression with APACHE II score was used to assess SNP association with overall disease severity.** Included in this table are the p-values of the logistic regression of ARDS patient genotype and APACHEII score by quartile. The APACHEII scores are split into quartiles and the 1^st^ and 4^th^ quartiles are used in a logistic regression against genotype using an additive model in the ARDS exome samples, TaqMan genotyped samples, and total ARDS samples. Regressions were also run on the stratified sub-populations of the ARDS patients. Associations were considered to be significant if P<0.05.
(DOCX)

Table S5 **A summary of the descriptive statistics for SNP rs78142040 in the exome sequenced ARDS, TaqMan genotyped ARDS patients, and total ARDS patients, where the controls are 1000 Genomes Project participants.** *, Chi-square tests were run on SNPs that were in both the controls and the cases; A, alternate allele; r, reference allele.
(DOCX)

Table S6 **A summary of the descriptive statistics for SNP rs9605146 in the exome sequenced ARDS, TaqMan genotyped ARDS patients, and total ARDS patients, where the controls are 1000 Genomes Project participants.** *, Chi-square tests were run on SNPs that were in both the controls and the cases; A, alternate allele; r, reference allele.
(DOCX)

Table S7 **A summary of the descriptive statistics for SNP rs3848719 in the exome sequenced ARDS, TaqMan genotyped ARDS patients, and total ARDS patients, where the controls are 1000 Genomes Project participants.** *, Chi-square tests were run on SNPs that were in both the controls and the cases; A, alternate allele; r, reference allele.
(DOCX)

Table S8 **A summary of the effect of the PCA adjustments on the genotypic trend test of the 3 SNPs.** 2 of the 3 SNPs were present in the filtered Caucasian ARDS+EUR controls population. PCA, principal components analysis; PCs, principal components; AA, African American ARDS; ASW, African Americans in the southwest 1000 Genomes Project; EA, European Ancestry or Caucasian; corr/trend, trend association test.
(DOCX)

Supporting Information S1 **The Supporting Information contains a detailed description of statistical methods used in this study.**
(DOCX)
