# Supplementary figures and images for: Identification of Novel Single Nucleotide Polymorphisms Associated with Acute Respiratory Distress Syndrome by Exome-Seq
Source: PLoS One. 2014 Nov 5;9(11):e111953. doi: 10.1371/journal.pone.0111953 (PMC4221189; doi:10.1371/journal.pone.0111953)

Shortt et al., Figure S1

**B**


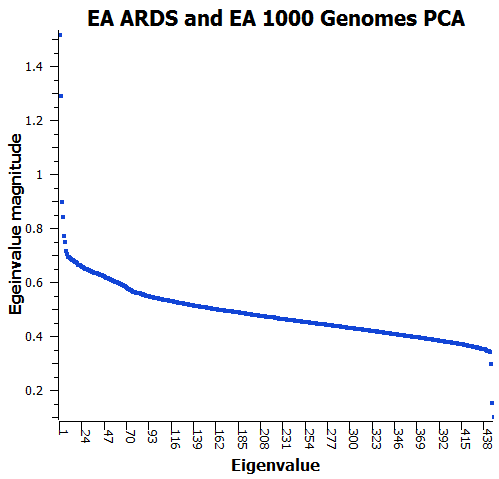

Supplement: Figure S1 — The scree plots of the eigenvalues generated by principal component analysis. The largest eigenvalues are used in corrections for population structure. (A) The All ARDS and ASW+EUR 1000 Genomes population eigenvalues. 535 principal components were measured and the largest eigenvalue is 7.32. (B) The Caucasian ARDS and EUR ARDS population eigenvalues. 449 principal components were measured and the largest eigenvalue is 1.52. (C) The African American ARDS and ASW 1000 Genomes population eigenvalues. 86 principal components were measured and the largest eigenvalue is 0.94. (DOCX) [file pone.0111953.s001.docx]
