# Supplementary material for: Identification of Novel Single Nucleotide Polymorphisms Associated with Acute Respiratory Distress Syndrome by Exome-Seq
Source: PLoS One. 2014 Nov 5;9(11):e111953. doi: 10.1371/journal.pone.0111953 (PMC4221189; doi:10.1371/journal.pone.0111953)
Supplement: Figure S2 — The quantile-quantile plots of genotypic trend test χ2 values for the African American ARDS and ASW 1000 Genomes population were derived using SVS v8.2.0. The straight line on each plot represents y = x. (A) QQ plot of expected χ2values versus the actual χ2values for the genotypic trend test of case-control status. The data are filtered on HWE, LD, and SNP call rate but not PCA corrected. (B) QQ plot of expected χ2values versus the actual χ2values for the genotypic trend test of case-control status. The data are corrected for the 2 largest principal components. (C) QQ plot of expected χ2values versus the actual χ2for the genotypic trend test of case-control status. The data have been filtered and corrected for 2 PCs and undergone sample outlier removal. (DOCX) [file pone.0111953.s002.docx]

Shortt et al., Figure S2
