# Supplementary material for: Identification of Novel Single Nucleotide Polymorphisms Associated with Acute Respiratory Distress Syndrome by Exome-Seq
Source: PLoS One. 2014 Nov 5;9(11):e111953. doi: 10.1371/journal.pone.0111953 (PMC4221189; doi:10.1371/journal.pone.0111953)
Supplement: Table S1 — A summary of the comparison groups used for the genetic association analysis. Association of the exome-seq SNPs with susceptibility was explored by comparing 96 ARDS patients to 440 controls from the 1000 Genomes Project. Analysis was stratified by race and etiology. (DOCX) [file pone.0111953.s003.docx]

Shortt et al., Table S1

**Table S1. Comparison groups for genetic association analysis.**

| Exome Seq Cases | Secondary Controls |
| --- | --- |
| 96 Cases ARDS: whole exome sequenced group | 440 EUR and ASW 1000 Genomes Project Controls |
| Cases stratified by race | 1000 Genomes Controls stratified by race |
| Cases stratified by ARDS etiology (Sepsis or Pneumonia) | 440 EUR and ASW 1000 Genomes Project Controls |
| Cases stratified by race and ARDS etiology (Sepsis or Pneumonia ) | 1000 Genomes Controls stratified by Race |

Association of the exome-seq SNPs with susceptibility was explored by comparing 96 ARDS patients to 440 controls from the 1000 Genomes Project. Analysis was stratified by race and etiology.
