# Supplementary material for: Identification of Novel Single Nucleotide Polymorphisms Associated with Acute Respiratory Distress Syndrome by Exome-Seq
Source: PLoS One. 2014 Nov 5;9(11):e111953. doi: 10.1371/journal.pone.0111953 (PMC4221189; doi:10.1371/journal.pone.0111953)
Supplement: Table S2 — A summary of the susceptibility χ2 tests for the 3 SNPs. The allelic chi-square test p-values for the exome sequenced ARDS patients ARDS patients and subgroups compared with the 1000 Genomes Project participants and subgroups, TaqMan genotyped ARDS patients and subgroups compared with the 1000 Genomes Project participants and subgroups, and the total ARDS patient population and subgroups compared with the 1000 Genomes Project participants and subgroups. P-values were considered to be significant if they were smaller than the Bonferroni corrected p-value of 2.95×10^−7^. (DOCX) [file pone.0111953.s004.docx]

Shortt et al., Table S2

**Table S2. Chi-Square test p-values for 3 SNPs in patients and controls.**

| SNP | rs78142040 | | |
| --- | --- | --- | --- |
| Gene | ARSD | | |
|  | 96 Exome | 117 TaqMan | Total 213 |
| ARDS χ^2^ P-value | 1.61E-41 | 3.05E-49 | 3.64E-47 |
| Caucasian χ^2^ P-value | 8.59E-32 | 8.65E-35 | 4.30E-34 |
| African American χ^2^ P-value | 4.69E-9 | 7.20E-11 | 3.36E-10 |
| Pneumonia χ^2^ P-value | 2.13E-31 | 2.85E-37 | 3.83E-35 |
| Sepsis χ^2^ P-value | 6.93E-51 | 4.89E-59 | 8.48E-57 |
| African American Sepsis χ^2^ P-value | 7.20E-12 | 7.11E-11 | 8.11E-12 |
| African American Pneumonia χ^2^ P-value | 2.06E-6 | 3.18E-10 | 5.58E-8 |
| Caucasian Sepsis χ^2^ P-value | 1.88E-37 | 1.60E-46 | 9.85E-43 |
| Caucasian Pneumonia χ^2^ P-value | 3.01E-25 | 3.30E-24 | 6.72E-25 |
| SNP | rs9605146 | | |
| Gene | XKR3 | | |
|  | 96 Exome | 117 TaqMan | Total 213 |
| ARDS χ^2^ P-value | 1.16E-51 | 1.13E-45 | 1.68E-59 |
| Caucasian χ^2^ P-value | 5.26E-34 | 7.31E-37 | 1.19E-44 |
| African American χ^2^ P-value | 1.86E-14 | 2.24E-8 | 4.52E-14 |
| Pneumonia χ^2^ P-value | 1.45E-40 | 1.22E-31 | 2.14E-47 |
| Sepsis χ^2^ P-value | 1.46E-36 | 1.09E-37 | 2.98E-49 |
| African American Sepsis χ^2^ P-value | 9.27E-11 | 3.56E-65 | 9.84E-11 |
| African American Pneumonia χ^2^ P-value | 1.38E-12 | 5.74E-7 | 9.94E-13 |
| Caucasian Sepsis χ^2^ P-value | 4.71E-25 | 6.52E-26 | 4.94E-35 |
| Caucasian Pneumonia χ^2^ P-value | 3.91E-24 | 2.82E-28 | 6.46E-36 |
| SNP | rs3848719 | | |
| Gene | ZNF335 | | |
|  | 96 Exome | 117 TaqMan | Total 213 |
| ARDS χ^2^ P-value | 3.44E-2 | 2.24E-1 | 7.80E-1 |
| Caucasian χ^2^ P-value | 4.24E-3 | 4.22E-1 | 2.78E-1 |
| African American χ^2^ P-value | 5.11E-2 | 6.61E-1 | 2.62E-1 |
| Pneumonia χ^2^ P-value | 1.65E-1 | 4.00E-1 | 8.78E-1 |
| Sepsis χ^2^ P-value | 8.67E-2 | 3.31E-1 | 7.85E-1 |
| African American Sepsis χ^2^ P-value | 9.96E-2 | 1.30E-1 | 7.80E-1 |
| African American Pneumonia χ^2^ P-value | 1.51E-1 | 4.05E-1 | 1.34E-1 |
| Caucasian Sepsis χ^2^ P-value | 3.76E-2 | 4.58E-1 | 4.26E-1 |
| Caucasian Pneumonia χ^2^ P-value | 3.57E-2 | 6.47E-1 | 3.99E-1 |

The allelic chi-square test p-values for the exome sequenced ARDS patients ARDS patients and subgroups compared with the 1000 Genomes Project participants and subgroups, TaqMan genotyped ARDS patients and subgroups compared with the 1000 Genomes Project participants and subgroups , and the total ARDS patient population and subgroups compared with the 1000 Genomes Project participants and subgroups. P-values were considered to be significant if they were smaller than the Bonferroni corrected p-value of 2.95x10^-7.
