# Supplementary material for: Identification of Novel Single Nucleotide Polymorphisms Associated with Acute Respiratory Distress Syndrome by Exome-Seq
Source: PLoS One. 2014 Nov 5;9(11):e111953. doi: 10.1371/journal.pone.0111953 (PMC4221189; doi:10.1371/journal.pone.0111953)
Supplement: Table S3 — Logistic regression with 60-day mortality from the day of diagnosis was used to assess SNP association with outcome. Included in this table are the p-values of the logistic regression of 60-day mortality against genotype using an additive model in the ARDS exome samples, TaqMan genotyped samples, and total ARDS samples. Associations were considered significant if p<0.05. (DOCX) [file pone.0111953.s005.docx]

Shortt et al.. Table S3

**Table S3. Logistic regression of genotype and 60-day mortality.**

| SNP | rs78142040 | | |
| --- | --- | --- | --- |
| Gene (s) | ARSD | | |
|  | 96 Exome | 117 TaqMan | Total 213 |
| ARDS p-value | 0.2630728 | 0.0339629 | 0.0173745 |
| Pneumonia p-value | 0.6602961 | 0.8575796 | 0.6581823 |
| Sepsis p-value | 0.5246962 | 0.028991 | 0.0348283 |
| Caucasian p-value | 0.2222798 | 0.9495742 | 0.408666 |
| African American p-value | 0.8547592 | na | 0.3606608 |
| African American Pneumonia p-value | 0.9282386 | na | 0.5363197 |
| African American Sepsis p-value | 0.4870023 | na | 0.596097 |
| Caucasian Sepsis p-value | 0.373584 | 0.8254455 | 0.4565353 |
| Caucasian Pneumonia p-value | 0.8171329 | 0.6294941 | 0.7551327 |
| SNP | rs9605146 | | |
| Gene (s) | XKR3 | | |
|  | 96 Exome | 117 TaqMan | Total 213 |
| ARDS p-value | 0.199116 | 0.1613938 | 0.8628899 |
| Pneumonia p-value | 0.0799541 | 0.1352877 | 0.9207455 |
| Sepsis p-value | 0.6639858 | 0.4980102 | 0.8681921 |
| Caucasian p-value | 0.676358 | 0.0857127 | 0.3488488 |
| African American p-value | 0.2706209 | 0.4411772 | 0.6577734 |
| African American Pneumonia p-value | 0.3251523 | 0.4184522 | 0.6835628 |
| African American Sepsis p-value | 0.553167 | 0.7626348 | 0.7754479 |
| Caucasian Sepsis p-value | 0.9381507 | 0.258087 | 0.527152 |
| Caucasian Pneumonia p-value | 0.4336663 | 0.1939841 | 0.4733411 |
| SNP | rs3848719 | | |
| Gene (s) | ZNF335 | | |
|  | 96 Exome | 117 TaqMan | Total 213 |
| ARDS p-value | 0.2983921 | 0.3583787 | 0.9136127 |
| Pneumonia p-value | 0.1851452 | 0.0323794 | 0.380281 |
| Sepsis p-value | 0.5992432 | 0.455294 | 0.3463909 |
| Caucasian p-value | 0.6700216 | 0.0122873 | 0.2039698 |
| African American p-value | 0.9736738 | na | 0.7412062 |
| African American Pneumonia p-value | 0.9149283 | na | 0.5810341 |
| African American Sepsis p-value | 0.8580141 | na | 0.8671714 |
| Caucasian Sepsis p-value | 0.919974 | 0.306832 | 0.5617649 |
| Caucasian Pneumonia p-value | 0.2672277 | 0.011951 | 0.2015335 |

Displayed are the p-values of the logistic regression of 60-day mortality against genotype conducted using an additive model in the ARDS exome samples, TaqMan genotyped samples, and total ARDS samples. Regressions were also run on the stratified sub-populations of the ARDS patients.
