# Supplementary material for: Identification of Novel Single Nucleotide Polymorphisms Associated with Acute Respiratory Distress Syndrome by Exome-Seq
Source: PLoS One. 2014 Nov 5;9(11):e111953. doi: 10.1371/journal.pone.0111953 (PMC4221189; doi:10.1371/journal.pone.0111953)
Supplement: Table S4 — Logistic regression with APACHE II score was used to assess SNP association with overall disease severity. Included in this table are the p-values of the logistic regression of ARDS patient genotype and APACHEII score by quartile. The APACHEII scores are split into quartiles and the 1st and 4th quartiles are used in a logistic regression against genotype using an additive model in the ARDS exome samples, TaqMan genotyped samples, and total ARDS samples. Regressions were also run on the stratified sub-populations of the ARDS patients. Associations were considered to be significant if P<0.05. (DOCX) [file pone.0111953.s006.docx]

Shortt et al., Table S4

**Table S4. Logistic regression of genotype and APACHEII score by quartile.**

| SNP | rs3848719 | rs9605146 | rs78142040 |
| --- | --- | --- | --- |
| Gene (s) | ZNF335 | XKR3 | ARSD |
| Exome samples |  |  |  |
| p-value | 0.10 | 0.15 | na |
| Odds Ratio | 0.50 | 2.00 | na |
| OR Lower Conf. Bound | 0.22 | 0.76 | na |
| OR Upper Conf. Bound | 1.16 | 5.26 | na |
| TaqMan samples |  |  |  |
| p-value | 0.30 | 0.36 | 0.06 |
| Odds Ratio | 0.68 | 0.70 | 2.60 |
| OR Lower Conf. Bound | 0.33 | 0.33 | 0.93 |
| OR Upper Conf. Bound | 1.42 | 1.50 | 7.26 |
| All samples |  |  |  |
| p-value | 0.03 | 0.78 | 0.29 |
| Odds Ratio | 0.55 | 1.08 | 1.51 |
| OR Lower Conf. Bound | 0.31 | 0.62 | 0.70 |
| OR Upper Conf. Bound | 0.96 | 1.89 | 3.25 |

The APACHEII scores are split into quartiles and the 1^st^ and 4^th^ quartiles are used in a logistic regression against genotype using an additive model in the ARDS exome samples, TaqMan genotyped samples, and total ARDS samples. Regressions were also run on the stratified sub-populations of the ARDS patients. Associations were considered to be significant if P<0.05.
