# Supplementary material for: Identification of Novel Single Nucleotide Polymorphisms Associated with Acute Respiratory Distress Syndrome by Exome-Seq
Source: PLoS One. 2014 Nov 5;9(11):e111953. doi: 10.1371/journal.pone.0111953 (PMC4221189; doi:10.1371/journal.pone.0111953)
Supplement: Table S5 — A summary of the descriptive statistics for SNP rs78142040 in the exome sequenced ARDS, TaqMan genotyped ARDS patients, and total ARDS patients, where the controls are 1000 Genomes Project participants. *, Chi-square tests were run on SNPs that were in both the controls and the cases; A, alternate allele; r, reference allele. (DOCX) [file pone.0111953.s007.docx]

Shortt et al., Table S5

**Table S5. rs78142040 statistics.**

| SNP | Rs78142040 | | |
| --- | --- | --- | --- |
| position | X:2832771 | | |
| Gene (s) | ARSD | | |
|  | 96 Exome | 117 TaqMan | Total 213 |
| χ^2^P-value* | 1.61E-41 | 3.05E-49 | 3.64E-47 |
| χ^2^ | 182.19 | 217.58 | 208.06 |
| Odds Ratio (Alternate Allele) | 444.02 | 544.49 | 498.09 |
| OR Lower Confidence Bound (Alt.) | 27.15 | 33.48 | 30.83 |
| OR Upper Confidence Bound (Alt.) | 7288.03 | 8855.41 | 8047.51 |
| Call Rate | 0.99 | 1.00 | 0.99 |
| Call Rate (Cases) | 0.94 | 1.00 | 0.97 |
| HWE P-value (Cases) | 1.77E-2 | 2.17E-2 | 1.18E-3 |
| HWE P-value (Controls) | 1 | 1 | 1 |
| HWE P-value | 0.42 | 0.56 | 0.47 |
| Number of Distinct Alleles | 2 | 2 | 2 |
| Alternate Allele | T | T | T |
| Alternate Allele Frequency | 0.03 | 0.05 | 0.07 |
| Alt. Allele Freq. (Cases) | 0.20 | 0.24 | 0.22 |
| Alt. Allele Freq. (Controls) | 0 | 0 | 0 |
| Reference Allele | C | C | C |
| Reference Allele Frequency | 0.97 | 0.95 | 0.93 |
| Ref. Allele Freq. (Cases) | 0.80 | 0.76 | 0.78 |
| Ref. Allele Freq. (Controls) | 1 | 1 | 1 |
| Genotype AA Count | 0 | 2 | 2 |
| AA (Cases) | 0 | 2 | 2 |
| AA (Controls) | 0 | 0 | 0 |
| Genotype Ar Count | 36 | 51 | 87 |
| Ar (Cases) | 36 | 51 | 87 |
| Ar (Controls) | 0 | 0 | 0 |
| Genotype rr | 494 | 504 | 558 |
| rr (Cases) | 54 | 64 | 118 |
| rr (Controls) | 440 | 440 | 440 |
| Alternate Allele A Count | 36 | 55 | 91 |
| A (Cases) | 36 | 55 | 91 |
| A (Controls) | 0 | 0 | 0 |
| Reference Allele r | 1024 | 1059 | 1203 |
| r (Cases) | 144 | 179 | 323 |
| r (Controls) | 880 | 880 | 880 |

A summary of the SNP rs78142040 in the exome sequenced ARDS, TaqMan genotyped ARDS patients, and total ARDS patients, where the controls are 1000 Genomes Project participants. *, Chi-square tests were run on SNPs that were in both the controls and the cases; A, alternate allele; r, reference allele.
