# Supplementary material for: Identification of Novel Single Nucleotide Polymorphisms Associated with Acute Respiratory Distress Syndrome by Exome-Seq
Source: PLoS One. 2014 Nov 5;9(11):e111953. doi: 10.1371/journal.pone.0111953 (PMC4221189; doi:10.1371/journal.pone.0111953)
Supplement: Table S6 — A summary of the descriptive statistics for SNP rs9605146 in the exome sequenced ARDS, TaqMan genotyped ARDS patients, and total ARDS patients, where the controls are 1000 Genomes Project participants. *, Chi-square tests were run on SNPs that were in both the controls and the cases; A, alternate allele; r, reference allele. (DOCX) [file pone.0111953.s008.docx]

Shortt et al., Table S6

**Table S6. rs9605146 statistics.**

| SNP | Rs9605146 | | |
| --- | --- | --- | --- |
| position | 22:17265194 | | |
| Gene (s) | XKR3 | | |
|  | 96 Exome | 117 TaqMan | Total 213 |
| χ^2^P-value* | 1.16E-51 | 1.13E-45 | 1.68E-59 |
| χ^2^ | 228.67 | 201.23 | 264.63 |
| Odds Ratio (Alternate Allele) | 17.06 | 13.77 | 15.16 |
| OR Lower Confidence Bound (Alt.) | 10.91 | 8.96 | 10.25 |
| OR Upper Confidence Bound (Alt.) | 26.66 | 21.18 | 22.41 |
| Call Rate | 0.99 | 1.00 | 1.00 |
| Call Rate (Cases) | 0.97 | 1.00 | 0.99 |
| HWE P-value (Cases) | 3.78E-1 | 8.22E-1 | 4.22E-1 |
| HWE P-value (Controls) | 3.72E-5 | 3.72E-5 | 3.72E-5 |
| HWE P-value | 1.13E-13 | 0.82 | 1.94E-12 |
| Number of Distinct Alleles | 2 | 2 | 2 |
| Alternate Allele | A | A | A |
| Alternate Allele Frequency | 0.11 | 0.11 | 0.15 |
| Alt. Allele Freq. (Cases) | 0.41 | 0.36 | 0.39 |
| Alt. Allele Freq. (Controls) | 0.04 | 0.04 | 0.04 |
| Reference Allele | G | G | G |
| Reference Allele Frequency | 0.90 | 0.89 | 0.85 |
| Ref. Allele Freq. (Cases) | 0.59 | 0.64 | 0.61 |
| Ref. Allele Freq. (Controls) | 0.96 | 0.96 | 0.96 |
| Genotype AA Count | 22 | 20 | 38 |
| AA (Cases) | 18 | 16 | 34 |
| AA (Controls) | 4 | 4 | 4 |
| Genotype Ar Count | 68 | 80 | 121 |
| Ar (Cases) | 41 | 53 | 94 |
| Ar (Controls) | 27 | 27 | 27 |
| Genotype rr | 443 | 457 | 491 |
| rr (Cases) | 34 | 48 | 82 |
| rr (Controls) | 409 | 409 | 409 |
| Alternate Allele A Count | 112 | 120 | 197 |
| A (Cases) | 77 | 85 | 162 |
| A (Controls) | 35 | 35 | 35 |
| Reference Allele r | 954 | 994 | 1103 |
| r (Cases) | 109 | 149 | 258 |
| r (Controls) | 845 | 845 | 845 |

A summary of the SNP rs9605146 in the exome sequenced ARDS, TaqMan genotyped ARDS patients, and total ARDS patients, where the controls are 1000 Genomes Project participants. *, Chi-square tests were run on SNPs that were in both the controls and the cases; A, alternate allele; r, reference allele.
