# Supplementary material for: Identification of Novel Single Nucleotide Polymorphisms Associated with Acute Respiratory Distress Syndrome by Exome-Seq
Source: PLoS One. 2014 Nov 5;9(11):e111953. doi: 10.1371/journal.pone.0111953 (PMC4221189; doi:10.1371/journal.pone.0111953)
Supplement: Table S7 — A summary of the descriptive statistics for SNP rs3848719 in the exome sequenced ARDS, TaqMan genotyped ARDS patients, and total ARDS patients, where the controls are 1000 Genomes Project participants. *, Chi-square tests were run on SNPs that were in both the controls and the cases; A, alternate allele; r, reference allele. (DOCX) [file pone.0111953.s009.docx]

Shortt et al., Table S7

**Table S7. rs3848719 statistics.**

| SNP | Rs3848719 | | |
| --- | --- | --- | --- |
| position | 20:44596545 | | |
| Gene (s) | ZNF335 | | |
|  | 96 Exome | 117 TaqMan | Total 213 |
| χ^2^P-value* | 3.44E-2 | 2.24E-1 | 7.80E-1 |
| χ^2^ | 4.47 | 1.48 | 0.08 |
| Odds Ratio (Alternate Allele) | 1.47 | 0.83 | 1.04 |
| OR Lower Confidence Bound (Alt.) | 1.03 | 0.61 | 0.81 |
| OR Upper Confidence Bound (Alt.) | 2.09 | 1.12 | 1.33 |
| Call Rate | 0.95 | 1 | 0.96 |
| Call Rate (Cases) | 0.74 | 1.00 | 0.88 |
| HWE P-value (Cases) | 3.81E-6 | 5.86E-1 | 2.86E-4 |
| HWE P-value (Controls) | 0.89 | 0.89 | 0.89 |
| HWE P-value | 0.04 | 0.69 | 0.03 |
| Number of Distinct Alleles | 2 | 2 | 2 |
| Alternate Allele | A | A | A |
| Alternate Allele Frequency | 0.40 | 0.38 | 0.39 |
| Alt. Allele Freq. (Cases) | 0.48 | 0.34 | 0.39 |
| Alt. Allele Freq. (Controls) | 0.39 | 0.39 | 0.39 |
| Reference Allele | G | G | G |
| Reference Allele Frequency | 0.60 | 0.62 | 0.61 |
| Ref. Allele Freq. (Cases) | 0.52 | 0.66 | 0.61 |
| Ref. Allele Freq. (Controls) | 0.62 | 0.62 | 0.62 |
| Genotype AA Count | 92 | 81 | 107 |
| AA (Cases) | 26 | 15 | 41 |
| AA (Controls) | 66 | 66 | 66 |
| Genotype Ar Count | 223 | 257 | 273 |
| Ar (Cases) | 16 | 50 | 66 |
| Ar (Controls) | 207 | 207 | 207 |
| Genotype rr | 196 | 219 | 248 |
| rr (Cases) | 29 | 52 | 81 |
| rr (Controls) | 167 | 167 | 167 |
| Alternate Allele A Count | 407 | 419 | 487 |
| A (Cases) | 68 | 80 | 148 |
| A (Controls) | 339 | 339 | 339 |
| Reference Allele r | 616 | 695 | 769 |
| r (Cases) | 74 | 154 | 228 |
| r (Controls) | 541 | 541 | 541 |

A summary of the SNP rs3848719 in the exome sequenced ARDS, TaqMan genotyped ARDS patients, and total ARDS patients, where the controls are 1000 Genomes Project participants. *, Chi-square tests were run on SNPs that were in both the controls and the cases; A, alternate allele; r, reference allele.
