# Supplementary material for: Identification of Novel Single Nucleotide Polymorphisms Associated with Acute Respiratory Distress Syndrome by Exome-Seq
Source: PLoS One. 2014 Nov 5;9(11):e111953. doi: 10.1371/journal.pone.0111953 (PMC4221189; doi:10.1371/journal.pone.0111953)
Supplement: Table S8 — A summary of the effect of the PCA adjustments on the genotypic trend test of the 3 SNPs. 2 of the 3 SNPs were present in the filtered Caucasian ARDS+EUR controls population. PCA, principal components analysis; PCs, principal components; AA, African American ARDS; ASW, African Americans in the southwest 1000 Genomes Project; EA, European Ancestry or Caucasian; corr/trend, trend association test. (DOCX) [file pone.0111953.s010.docx]

Shortt et al., Table S8

**Table S8. Effect of PCA on the genotypic trend test of 3 SNPs.**

|  |  | rs3848719 | rs9605146 | rs78142040 |
| --- | --- | --- | --- | --- |
| ARDS and ASW+ EA 1000 Genomes | Passed filtering? | no | no | no |
| no PCA | corr/trend p-value | 4.29E-2 | 1.90E-39 | 6.86E-43 |
| corrected for PCs | corr/trend p-value | NA | NA | NA |
| corrected for PCs with outlier removal on PCs | corr/trend p | NA | NA | NA |
| AA ARDS and ASW1000 Genomes | passed filtering? | no | no | no |
| no PCA | corr/trend p-value | 7.91E-2 | 1.08E-10 | 1.22E-9 |
| corrected for 2 PCs | corr/trend p-value | NA | NA | NA |
| corrected for 2 PCs with outlier removal on 2 PCs | corr/trend p-value | NA | NA | NA |
| EA ARDS and EA 1000 Genomes | passed filtering? | no | yes | yes |
| no PCA | corr/trend p-value | 4.76E-03 | 5.67E-27 | 1.52E-32 |
| corrected for 6PCs | corr/trend p-value | NA | 2.62E-13 | 1.19E-11 |
| corrected for 6 PCs with outlier removal on 6 PCs | corr/trend p-value | NA | 2.17E-19 | 2.62E-04 |

PCA, principal components analysis; PCs, principal components; AA, African American ARDS; ASW, African Americans in the southwest 1000 Genomes Project; EA, European Ancestry or Caucasian; corr/trend, trend association test
