# Supplementary material for: Identification of Novel Single Nucleotide Polymorphisms Associated with Acute Respiratory Distress Syndrome by Exome-Seq
Source: PLoS One. 2014 Nov 5;9(11):e111953. doi: 10.1371/journal.pone.0111953 (PMC4221189; doi:10.1371/journal.pone.0111953)
Supplement: Supporting Information S1 — The Supporting Information contains a detailed description of statistical methods used in this study. (DOCX) [file pone.0111953.s011.docx]

Shortt et al. Supporting Information S1

1. **Supporting Information S1**
   1. **SNPs associated with ARDS susceptibility:** To conduct the statistical analyses the ARDS population VCF files were imported to SNP & Variation Suite 8 (SVS v8.2.0). The 1000 genomes exome sequence data were provided by Golden Helix in a *.dsf file. These files and their phenotype data were joined and used to calculate the χ^2^ values and χ^2^p-values for the SNPs that were matched by location using a basic allelic statistical model. Calculations were also stratified by ancestry and ARDS comorbidity (sepsis and pneumonia). 1,382,399 SNPs were identified in the ARDS patients and 714,074 SNPs were available from the 1000 Genomes Project. The Bonferroni corrected p-value was determined to be 2.95x10^-7^ because 169,376 SNPs were matched between the 1000 Genomes data and the ARDS patient data. SNPs that were present in all 4 of the ARDS sub populations (Caucasians with sepsis, Caucasians with pneumonia, African Americans with sepsis, and African Americans with pneumonia) were selected for the statistical association tests, totaling 49,834. SNPs with a chi square p-value<2.95x10^-7^ in at least 2 of the 5 main populations (All ARDS vs. 1000 Genomes, All Sepsis vs. 1000 Genomes, All Pneumonia vs. 1000 Genomes, African American ARDS vs. ASW 1000 Genomes, and Caucasian ARDS vs. EUR 1000 Genomes) were considered further. The χ^2^ test of the categorical data were used to identify novel targets for study. This was represented graphically using Manhattan plots generated using SVS v8.2.0.
   2. **Hardy-Weinberg equilibrium analysis of SNPs:** Hardy-Weinberg equilibrium and SNP call rate were calculated using the Marker Statistics feature of SVS v8.2.0. SNPs were considered to be in HWE if the p-value was >1×10^-4^. The call rate of the 1000 genomes project controls was 100%, meaning there were no missing genotypes. The call rate of the ARDS SNPs was considered to be good if it was >95% in the total exome sequenced population [24]. This data were compiled and used to determine the top SNP candidates using the criteria that they were present in all 4 individual ARDS populations (African American Sepsis, African American Pneumonia, Caucasian Sepsis, and Caucasian Pneumonia), had a χ^2^ p-value < 0.01 in the 5 main ARDS populations, had a χ^2^<2.95x10^--7^ in at least 2 of the 5 main populations, and had some association with severity or outcome. Functional prediction information was also taken into account, and nonsynonymous SNPs were preferred because of their likelihood of causing direct functional effects using Provean, Sift, and Polyphen2 (http://provean.jcvi.org, http://genetics.bwh.harvard.edu/pph2).
   3. **Population structural analysis**: population structure differences between the ARDS exome sequenced patients and the 1000 Genomes health controls were visualized using QQ plots of the –log10 trend test χ^2^ values and by computing the principal components using the Eigensoft PCA method in SVS for the All ARDS and All 1000 Genomes group, Caucasian ARDS and EUR 1000 Genomes subgroup, and African American ARDS and ASW 1000 Genomes subgroup [26-28]. The exome data were first filtered to include only SNPs that were in Hardy- Weinberg equilibrium (alleles distributed as expected) in the control samples (HWE p>0.0001). SNPs that had more than 2 alleles or had a call rate of <0.95 in the combined cases and controls were omitted. SNPs that were in linkage disequilibrium (R^2^<0.2 in 200 SNP window moving in 25 SNP increments) were omitted from analysis. PCA was conducted on the filtered data individually for the ARDS and 1000 Genomes population (ASW plus EUR) and the ancestry subgroups (Caucasian ARDS and EUR 1000 Genomes, and African American ARDS and ASW 1000 Genomes). The number of components used for correction was determined by examining the Scree plots and logistic regression of the principal components with case-control status. Outlying samples were identified as being more than 6 SD away from the number of PCs chosen for correction in a genotype trend test and the PCs were recomputed 5 times [30]. The genomic inflation factors (λ) were calculated for the trend test for the unfiltered exome cases vs. controls, filtered but pre-PCA data, and PCA corrected data (with and without outlier removal) to quantify the amount of error that could be attributed to population stratification or genotyping error [29, 40]. This analysis was conducted using SVS v8.2.0.
